# Medical Expenditures Attributed to Asthma and Chronic Obstructive Pulmonary Disease Among Workers — United States, 2011–2015

**DOI:** 10.15585/mmwr.mm6926a1

**Published:** 2020-07-03

**Authors:** Girija Syamlal, Anasua Bhattacharya, Katelynn E. Dodd

**Affiliations:** ^1^Respiratory Health Division, National Institute for Occupational Safety and Health, CDC; ^2^Office of the Director, National Institute for Occupational Safety and Health, CDC.

Asthma and chronic obstructive pulmonary disease (COPD) are respiratory conditions associated with a significant economic cost among U.S. adults (*1*,*2*), and up to 44% of asthma and 50% of COPD cases among adults are associated with workplace exposures (*3*). CDC analyzed 2011–2015 Medical Expenditure Panel Survey (MEPS) data to determine the medical expenditures attributed to treatment of asthma and COPD among U.S. workers aged ≥18 years who were employed at any time during the survey year. During 2011–2015, among the estimated 166 million U.S. workers, 8 million had at least one asthma-related medical event,* and 7 million had at least one COPD-related medical event. The annualized total medical expenditures, in 2017 dollars, were $7 billion for asthma and $5 billion for COPD. Private health insurance paid for 61% of expenditures attributable to treatment of asthma and 59% related to COPD. By type of medical event, the highest annualized per-person asthma- and COPD-related expenditures were for inpatient visits: $8,238 for asthma and $27,597 for COPD. By industry group, the highest annualized per-person expenditures ($1,279 for asthma and $1,819 for COPD) were among workers in public administration. Early identification and reduction of risk factors, including workplace exposures, and implementation of proven interventions are needed to reduce the adverse health and economic impacts of asthma and COPD among workers.

MEPS is an annual household survey administered to a nationally representative sample of the noninstitutionalized civilian U.S. population through an in-person interview.^†^ During the study period, 2011–2015, the years with the most recent available data, the annual survey response rates ranged from 54.9% in 2011 to 47.7% in 2015. To improve the precision and reliability of estimates, 2011–2015 data were combined.

Participants’ self-reported information on medical conditions, the associated medical events, payments, source of payments, and employment status were collected during the MEPS interview. MEPS professional coders assigned a code to the medical condition or conditions associated with each medical event reported by the participant, using the *International Classification of Diseases, Ninth Revision, Clinical Modification* (ICD-9-CM). Each medical event could be assigned one or more ICD-9-CM codes. Medical events associated with treated asthma were identified using ICD-9-CM code 493 and medical events associated with treated COPD were identified using ICD-9-CM codes 490, 491, 492, and 496.^§^

Expenditures were calculated from the sum of payments from Medicaid, Medicare, private insurance, out-of-pocket expenses, and other sources^¶^ for each treated asthma- and COPD-associated medical event. The annualized, total and per-person unadjusted medical expenditures for workers with asthma and COPD were estimated by type of medical event and source of payments. Workers were those who were “currently employed,” “had no job at the interview date but had a job to return to” or were employed at any time during the survey year. Information on participants’ current industry was categorized into 15 industry groups.**

Data were weighted to produce nationally representative estimates using sample weights adjusted for the 5-year data. Data were analyzed using SAS software (version 9.4; SAS Institute) to account for the complex survey design. Estimates with relative standard error (standard error of the estimate divided by the estimate) ≥30% are not reported. All expenditure values were expressed in 2017 U.S. dollars using the Medical Care Consumer Price Index.^††^

During 2011–2015, among the annual average estimated 166 million U.S. persons aged ≥18 years who were working at any time during the survey year, 8 million (5%) workers had at least one asthma-related medical event, and 7 million (4%) had at least one COPD-related medical event, which accounted for 21 million asthma-associated and 15 million COPD-related medical events (Table 1). The proportion of current smokers among workers who had an asthma event during the study period was 13%; 24% had a COPD event. Annualized average per-person medical expenditures attributable to treated asthma and COPD were $901 and $681, respectively. Highest annualized expenditures per person attributable to treated asthma and treated COPD were among non-Hispanic whites ($923 and $742, respectively), persons with health insurance ($914 and $705, respectively), and current nonsmokers ($936 and $692, respectively). By age group, annualized per-person expenditures for asthma and COPD were highest among persons aged 45–64 years ($1,081) ≥65 years ($1,090), respectively.

**TABLE 1:** Estimated number of workers with an asthma-related or chronic obstructive pulmonary disease–related medical event and annualized total and per-person expenditures,* by selected characteristics among workers aged ≥18 years — Medical Expenditure Panel Survey, United States, 2011–2015

Characteristic^†^	No. of workers (x1,000)	Asthma			Chronic obstructive pulmonary disease		
		No. of workers with an event (x1,000)	Total expenditures ($) in millions	Average expenditure ($) per person	No. of workers with an event (x1,000)	Total expenditures ($) in millions	Average expenditure ($) per person
**Total**	**166,347**	**7,920**	**7,137**	**901**	**7,371**	**5,021**	**681**
**Age group (yrs)**							
18–34	21,704	1,012	626	619	499	93	186
35–44	70,773	2,961	2,268	766	2,421	515	213
45–64	63,467	3,375	3,648	1,081	3,568	3,355	940
≥65	10,403	659	595	903	971	1,058	1,090
**Sex**							
Men	86,749	2,954	2,473	837	3,057	2,238	732
Women	79,598	5,053	4,663	923	4,403	2,783	632
**Race/Ethnicity**							
Hispanic	26,499	891	745	836	594	129	217
White, non-Hispanic	107,676	5,564	5,140	923	5,865	4,350	742
Black, non-Hispanic	18,712	1,037	879	847	613	375	611
Other	13,460	515	372	722	388	168	433
**Household income **							
<$35,000	39,521	1,794	1,520	847	1,810	1,091	603
$35,000–$74,999	53,373	2,486	2,112	850	2,579	2,113	819
≥$75,000	73,375	3,726	3,505	940	3,070	1,817	592
**Education**							
Less than high school	67,266	2,396	2,185	911	2,961	2,838	959
High school or more	98,269	5,607	4,951	883	4,468	2,170	486
**Insurance coverage**							
Yes	142,396	7,509	6,866	914	6,916	4,875	705
No	23,951	498	270	542	544	146	268
**U.S. Census region** ^§^							
Northeast	29,696	1,851	1,787	965	1,281	984	768
Midwest	36,660	1,757	1,621	923	1,941	1,757	905
South	60,870	2,683	2,381	887	2,826	1,117	395
West	38,809	1,714	1,348	787	1,408	1,162	825
**Current smoking status** ^¶^							
Smoker	24,820	955	664	695	1,636	1,024	626
Nonsmoker	125,570	6,514	6,097	936	5,220	3,612	692

* All medical expenditures expressed in 2017 U.S. dollars.

^†^ Missing information on education for 812,000; on household income for 78,000; on region for 312,000; and on smoking status for 15,957,000 workers. Columns do not sum to totals because of rounding; those with missing values were excluded from the analysis.

^§^ https://www2.census.gov/geo/pdfs/maps-data/maps/reference/us_regdiv.pdf.

^¶^ Based on yes/no responses to the question “Do you currently smoke?”

Prescription medication accounted for the highest number of events for asthma (15 million) and for COPD (8 million) (Table 2). The total annualized medical expenditures for treated asthma-related medical events among workers were $7 billion, and they were $5 billion for COPD. Derived using the pooled population-attributable fraction of 16% for asthma and 14% for COPD (*3*), annualized expenditures attributable to workplace exposures exceeded $1 billion for asthma and $700 million for COPD.

**TABLE 2:** Estimated number of workers with asthma-related or chronic obstructive pulmonary disease–related medical event and annualized total and per-person expenditures,* by type of event and source of payment — Medical Expenditure Panel Survey, United States, 2011–2015

Event/Source of payment^†^	Asthma				Chronic obstructive pulmonary disease			
	Total no. of events	No. of workers with an event (x1,000)	Total expenditures ($) in millions	Average expenditure ($) per person	Total no. of events	No. of workers with an event (x1,000)	Total expenditures ($) in millions	Average expenditure ($) per person
**Total** ^§^	**21,206**	**7,920**	**7,137**	**901**	**14,540**	**7,371**	**5,021**	**681**
**Type of event**								
Prescription drugs	15,008	5,361	5,216	973	8,421	3,733	1,627	436
Office based visits	5,503	2,117	921	435	5,262	3,064	1,041	340
Inpatient visits	66	63	519	8,238	71	62	1,711	27,597
Emergency department visits	412	332	372	1,121	441	375	442	1,178
Outpatient visits	210	126	106	841	293	205	166	810
Home health visits	8	8	3	375	52	21	35	1,667
**Source of payment**								
Private insurance	16,917	5,331	4,326	811	9,235	4,173	2,949	707
Out of pocket^¶^	22,907	6,673	1,370	205	14,489	5,993	664	111
Medicaid	3,011	977	681	697	1,859	647	391	604
Medicare	2,473	635	446	702	2,399	775	761	983
Other**	2,109	583	314	556	1,437	592	256	432

* All medical expenditures expressed in 2017 U.S. dollars.

**^†^** More than one type of medical event and source of payment could be reported per person.

^§^ Columns do not sum to totals because of rounding.

^¶^ Portion of total payments made by persons or families for services received during the year, including deductibles, coinsurance, and copayments for covered services plus all expenditures for services not covered by the insurance.

** Includes payments from the Department of Veterans Affairs (excluding TRICARE); other federal sources (Indian Health Service, military treatment facilities, and other care provided by the Federal Government); various state and local sources (community and neighborhood clinics, state and local health departments, and State programs other than Medicaid); payments from Workers' Compensation; and, other unclassified sources (e.g., automobile, homeowner's, or liability insurance, and other miscellaneous or unknown sources). It also includes private insurance payments reported for persons without private health insurance coverage during the year, as defined in the Medical Expenditure Panel Survey, and Medicaid payments reported for persons who were not enrolled in the Medicaid program at any time during the year (https://meps.ahrq.gov/mepstrends/hc_cond/).

By type of medical event, prescription drugs for asthma ($5 billion) and inpatient visits for COPD ($2 billion) accounted for the highest total annualized expenditures. Annualized expenditures per person were highest for inpatient visits (excluding prescription medications): $8,238 for asthma and $27,597 for COPD. By source of payment, private health insurance paid for 61% ($4 billion) of expenditures attributable to treated asthma and 59% ($3 billion) of expenditures attributable to treated COPD. The highest annualized expenditures per person were paid by private insurance for asthma ($811) and Medicare for COPD ($983).

Among industry groups, the annualized expenditures per person for treated asthma were highest among public administration workers ($1,279), followed by transportation and utilities workers ($1,222) (Table 3). The annualized expenditures per person for treated COPD were highest among public administration workers ($1,819), followed by construction workers ($1,198).

**TABLE 3:** Estimated number of workers with an asthma-related or chronic obstructive pulmonary disease–related medical event and annualized total and per-person expenditures,* by industry groups among workers aged ≥18 years payment — Medical Expenditure Panel Survey, United States, 2011–2015

Industry group	No. of workers (x1,000)	Asthma			Chronic obstructive pulmonary disease		
		No. of workers with an event (x1,000)	Total expenditures ($) in millions	Average expenditure ($) per person	No. of workers with an event (x1,000)	Total expenditures ($) in millions	Average expenditure ($) per person
Natural resources	2,320	57	47	825	96	36	375
Mining	792	40	46	1,150	—^†^	—	—
Construction	10,500	221	214	968	344	412	1,198
Manufacturing	16,354	658	733	1,114	874	614	703
Wholesale and retail trade	21,400	1,005	940	935	821	404	492
Transportation and utilities	7,771	284	347	1,222	349	155	444
Information	3,306	155	136	877	137	76	555
Financial activities	10,142	435	363	834	416	180	433
Professional and business services	19,592	957	773	808	806	327	406
Education health and social services^§^	38,507	2,421	2,250	929	2,004	1,435	716
Leisure and hospitality	14,492	691	555	803	552	383	694
Other services^¶^	8,515	363	324	893	398	199	500
Public administration^§^	8,247	535	684	1,279	469	853	1,819
Military	355	—	—	—	—	—	—
Unclassifiable/Missing	4,054	—	—	—	—	—	—

*All medical expenditures expressed in 2017 U.S. dollars.

^†^ Unreliable estimates (relative standard error >30; standard error of the estimate divided by the estimate), data suppressed.

^§^ Includes education services workers and ambulatory healthcare services workers, hospitals, nursing and residential care facility workers and social assistance.

^§^ https://datausa.io/profile/naics/92.

^¶^ Other services industries include repair and maintenance, personal and laundry services, religious, grantmaking, civic, professional services, and private households and similar organizations.

## Discussion

COPD and asthma combined were among the top five most costly medical conditions among U.S. adults in 2012 (*4*). Among workers, the total medical expenditures attributable to the treatment of asthma and COPD were substantial ($7 billion for asthma and $5 billion for COPD) and varied by sociodemographic characteristics and industry. Workers in the public administration industry (e.g., police officers, correctional officers, jailers, firefighters, and secretaries and administrative assistants)^§§^ had the highest annualized per-person expenditures for both asthma and COPD. In the public administration industry, an estimated 7.4% of workers have asthma, and 3.5% of workers have COPD.^¶¶^ Variation in expenditures by industry might reflect the differences in prevalences, health insurance status, and access to medical care. Overall, workers with no health insurance had lower medical expenditures for asthma and for COPD than did those who had health insurance, suggesting that the uninsured population might have sought services through free clinics or might have limited their care-seeking (*1**,**3*). Based on the 2019 pooled population attributable fraction estimates of 16% for asthma and 14% for COPD, the estimated expenditures attributable to workplace exposures among workers exceeded $1 billion for asthma and $700 million for COPD. 

Among workers, prescription medications accounted for the highest proportion of total medical expenditures attributable to the treatment of asthma, as did inpatient visits for the treatment of COPD, similar to previous findings among all U.S. adults (*1*,*5*). Inpatient visits accounted for the highest per-person expenditure for treated asthma and COPD. Higher expenditures related to inpatient visits have been highly correlated with asthma and COPD exacerbation severity (*5*,*6*). An estimated 67% of total asthma-attributable medical expenditures were associated with prescription medications, which is higher than the 51% observed previously among all U.S. adults (*1*). The higher prescription medication expenditures might be associated with new and more costly treatment options or could be a result of inflation adjustments (*1*,*7*,*8*). Moreover, workers are more likely to have health insurance than are nonworkers (*9*); therefore, they might have fewer financial barriers to purchasing prescription medications, which might also partially explain the higher expenditures among workers.

The findings in this report are subject to at least four limitations. First, the number of medical events and expenditures associated with asthma and COPD were self-reported by respondents and might be subject to recall bias. However, self-reported medical events and expenditure data, including office-based visits, emergency department visits, and hospitalizations, have been shown to correspond well with health care utilization data (*10*). Second, workers could have been treated for comorbidities during their asthma- or COPD-related medical encounter; therefore, a portion of medical expenditures might not be directly associated with asthma or COPD. Third, workers might have changed employment from the industry in which they were employed at the time of their asthma- or COPD-related medical events; therefore, medical expenditures by industry group might not reflect the actual industry the worker was employed in when the expenditure was incurred. Finally, small sample sizes for some groups resulted in unreliable estimates.

Annualized overall and per-person medical expenditures attributable to treated asthma and treated COPD among workers were substantial. Early identification and reduction of risk factors, including workplace exposures (e.g., vapors dusts gas and fumes), and implementation of proven interventions are needed to reduce the adverse health and economic impacts of asthma and COPD among workers. Prioritizing intervention efforts aimed at preventing asthma and COPD among workers, especially among those with higher medical costs, by supporting workplace programs and policies (e.g., smoke-free workplace policies, smoking cessation programs, and workplace exposure control measures) can reduce the impact of disease and improve worker health.*** Continued surveillance is important to identify workers with high prevalences of asthma or COPD and less consistent access to health care.

**Summary**

**What is already known about this topic?**

Asthma and chronic obstructive pulmonary disease (COPD) are associated with substantial economic and health costs among U.S. workers.

**What is added by this report?**

During 2011–2015, total annualized medical expenditures among U.S. workers were $7 billion ($901 per person) for asthma and $5 billion ($681 per person) for COPD. Inpatient visits were associated with the highest average per-person expenditures for both conditions. Insured workers incurred higher expenditures than did uninsured workers.

**What are the implications for public health practice?**

Early identification and reduction of risk factors, including workplace exposures (e.g., vapors, gas, dusts, and fumes), and implementation of proven interventions are needed to reduce the adverse health and economic impacts of asthma and COPD among workers.

## Footnotes

* Hospital inpatient care, outpatient visits, emergency department visits, office-based visits, home health care, or purchase of prescribed medicines.

† https://meps.ahrq.gov/mepsweb/survey_comp/household.jsp.

§ https://meps.ahrq.gov/data_stats/download_data/pufs/h180/h180doc.pdf.

¶ Veterans Administration/CHAMPVA, TRICARE, and other federal sources include Indian Health Service, military treatment facilities, and other care by the federal government. Other state and local sources include community and neighborhood clinics, state and local health departments, and state programs other than Medicaid, and workers’ compensation. Other unclassified sources include sources such as automobile, homeowner’s, and liability insurance and other miscellaneous or unknown sources.

** https://meps.ahrq.gov/data_stats/download_data/pufs/ind_occ/ind3.pdf.

†† https://www.in2013dollars.com/Medical-care/price-inflation/2015-to-2017?amount.

§§ https://datausa.io/profile/naics/92/.

¶¶ https://wwwn.cdc.gov/eWorld/Set/Work-Related_Respiratory_Diseases/88; https://www.cdc.gov/mmwr/volumes/67/wr/mm6713a1.htm.

*** https://goldcopd.org/wp-content/uploads/2018/11/GOLD-2019-v1.7-FINAL-14Nov2018-WMS.pdf; https://ginasthma.org/wp-content/uploads/2019/06/GINA-2019-main-report-June-2019-wms.pdf.
